# Machine Learning–Based Prognostic Models for Functional Outcomes in Spinal Cord Injury: Systematic Review

**DOI:** 10.2196/84980

**Published:** 2026-06-23

**Authors:** Yuan Liu, Xiangxia Meng, Yi Ding, Ruifa Yao, Shuchang Xu

**Affiliations:** 1 College of Nursing Shandong University of Traditional Chinese Medicine Jinan China; 2 Healthcare Office The Second Affiliated Hospital of Shandong University of Traditional Chinese Medicine Jinan China; 3 Department of Rehabilitation Medicine The Second Affiliated Hospital of Shandong University of Traditional Chinese Medicine Jinan China

**Keywords:** spinal cord injury, machine learning, prognosis, prediction model, risk of bias, systematic review

## Abstract

**Background:**

Machine learning is increasingly used to develop prognostic prediction models for spinal cord injury. Nevertheless, current studies exhibit heterogeneity in outcome measures, predictors, modeling strategies, and validation methods. Moreover, the reporting quality, risk of bias, and clinical applicability of these models have not been systematically evaluated using assessment tools specific to prediction models.

**Objective:**

This review aimed to assess the reporting quality and risk of bias of machine learning–based prognostic models for spinal cord injury, and evaluate their clinical applicability, model features, validation, and implementation barriers.

**Methods:**

We searched the China National Knowledge Infrastructure (CNKI), Wanfang Data, VIP Database, Sinomed, PubMed, Web of Science, Embase, and Scopus databases from their inception up to May 14, 2025. Two investigators independently screened studies, extracted data, and assessed risk of bias. Reporting quality and risk of bias were evaluated using the Transparent Reporting of a Multivariable Prediction Model for Individual Prognosis or Diagnosis (TRIPOD) statement and the Prediction Model Risk of Bias Assessment Tool (PROBAST). Descriptive statistics and narrative synthesis were used to summarize the findings.

**Results:**

In total, 19 cohort studies were included. TRIPOD adherence ranged from 54.8% (17/31) to 81.1% (30/37), with a median of 74.2% (IQR 64.5%-77.4%). Overall, all 19 studies were judged to have a high risk of bias, mainly because of limitations in the analysis domain. Only 1 (5.3%) study included external validation, while 16 (84.2%) studies used internal validation and 2 (10.5%) studies reported model development only. No study justified the sample size; 6 (31.6%) studies reported imputation or other methods for handling missing data, and calibration was rarely reported.

**Conclusions:**

Machine learning shows potential for spinal cord injury prognostic modeling, especially when complex clinical or imaging data are available. However, existing evidence is limited by incomplete reporting, high risk of bias, substantial heterogeneity, and limited external validation. Larger, methodologically robust studies with standardized outcomes, external validation, and evaluation of clinical usefulness are necessary before these models can be implemented in routine clinical practice.

**Trial Registration:**

PROSPERO CRD420251071502; https://www.crd.york.ac.uk/PROSPERO/view/1071502

## Introduction

### Background

Spinal cord injury (SCI) is a serious central nervous system disorder caused by trauma, tumors, or inflammation. It results in varying degrees of motor and sensory impairments, along with autonomic nervous system dysfunction [1,2]. Globally, SCI affects 40 to 80 individuals per million, mainly male individuals aged 15 to 35 years [3,4]. Treatment options include surgery, medications, new technologies, and rehabilitation with assistive devices [1,5-8]. However, differences in injury severity, neurological symptoms, treatments, and recovery make individualized prognosis challenging [9,10]. Therefore, reliable prediction models are crucial for early risk assessment, goal setting, rehabilitation planning, and communication with patients and families [11,12].

Machine learning (ML), a branch of artificial intelligence (AI), develops predictive models by identifying inherent patterns in clinical data. Its significance in supporting clinical decisions has led to cross-disciplinary research endeavors [13,14]. Recently, ML models have been developed to predict outcomes in patients with SCI, including neurological recovery, functional independence, walking ability, daily activity performance, and postoperative recovery [15-17]. The included studies show considerable variation in outcome measures, such as the American Spinal Injury Association Impairment Scale (AIS) [18], Spinal Cord Independence Measure (SCIM) [19], Functional Independence Measure (FIM) [20], Functional Ambulation Category at discharge (FAC-DC) [21], and activities of daily living (ADL) [22]. The modeling approaches also varied greatly, from logistic regression [23] and support vector machines [24] to random forests, Extreme Gradient Boosting (XGBoost) [18], Light Gradient Boosting Machine (LightGBM) [25], convolutional neural networks [26], and ensemble methods. This diversity indicates considerable methodological innovation but also makes it challenging to compare models across studies and assess their clinical applicability.

Although ML has been widely used for SCI diagnosis and prognosis, its practical application encounters some limitations. The variability in patient characteristics, treatment approaches, and imaging features presents challenges in creating reliable neurofunctional outcome prediction models [15]. Furthermore, differences in prediction targets and the lack of standardized evaluation methods hinder effective model comparison. In 2024, Habibi et al [27] conducted a systematic review on ML in SCI that covered diagnostic, prognostic, and management studies. Of the 21 included studies, 5 focused on diagnostic accuracy, whereas 16 addressed prognostic factors or management strategies. In addition, methodological quality was assessed using the Quality Assessment of Diagnostic Accuracy Studies 2 (QUADAS-2), which is intended for diagnostic accuracy studies rather than prediction model research [28]. Therefore, many studies included in that review were not eligible for this review, which focused specifically on cohort-based prognostic prediction models for functional outcomes in SCI. As a result, important issues relevant to prognostic prediction models—such as reporting completeness, risk of bias in model development and validation, handling of missing data, sample size adequacy, distinction between internal and external validation, and implications for model applicability—were not sufficiently evaluated.

### Aims

Therefore, a targeted review of ML-based prognostic prediction models for SCI is needed. This systematic review aimed to evaluate the reporting quality and risk of bias of existing ML-based prognostic prediction models for SCI using Transparent Reporting of a Multivariable Prediction Model for Individual Prognosis or Diagnosis (TRIPOD) and Prediction Model Risk of Bias Assessment Tool (PROBAST) and secondarily to examine their clinical applicability, model characteristics, validation strategies, and barriers to clinical implementation.

## Methods

### Study Design and Registration

This review was conducted in accordance with the PRISMA (Preferred Reporting Items for Systematic Reviews and Meta-Analyses) statement [29]. The study protocol was registered prospectively with PROSPERO, the International Prospective Register of Systematic Reviews, under registration number CRD420251071502.

### Search Strategy

Systematic searches were performed in the China National Knowledge Infrastructure (CNKI), Wanfang Data, VIP Database, Chinese Biomedical Literature Service System (Sinomed), PubMed, Web of Science, Embase, and Scopus databases. The search strategy combined MeSH (Medical Subject Headings) terms and free-text terms such as “Spinal Cord Injuries/Myelopathy, Traumatic/Cord Traumas, Spinal/Post Traumatic Myelopathy,” “Machine Learning/Learning, Deep/Artificial Intelligence/Machine Intelligence,” and “predictive model/prediction/forecasting/risk assessment/risk prediction/models.” The searches covered all records from each database’s start date up to May 14, 2025.

### Eligibility Criteria and Study Selection

Two trained reviewers independently screened records and full texts according to predefined criteria. Screening decisions were cross-verified, and disagreements were resolved by discussion with a third reviewer.

Eligible studies met the following criteria: (1) population: individuals diagnosed with SCI who were aged 18 years or older, regardless of treatment methods (eg, surgery or rehabilitation) [30]; (2) intervention: development, validation, or both of prognostic models; (3) outcomes: metrics assessing the model’s predictive validity and accuracy; (4) study designs: cohort studies, including retrospective and prospective studies; (5) language: publications in Chinese or English. Studies were excluded if they only analyzed prognostic factors without building a prediction model, provided insufficient details on model development, or were nonoriginal publications, such as reviews, systematic reviews, meta-analyses, case reports, commentaries, or conference abstracts.

### Data Extraction and Model Characteristics

A structured data extraction form was developed following the CHARMS (Critical Appraisal and Data Extraction for Systematic Reviews of Prediction Modeling Studies) checklist for systematic reviews of prediction models [31] and subsequently verified for accuracy. The included studies provided the following information: author, publication year, country, study design, outcome incidence, data source, predictors, model type, sample size, predictor selection methods, key predictors, missing data handling techniques, modeling approaches, model presentation format, performance metrics, and validation type (development only, internal validation, or external validation). Two reviewers independently extracted the data, cross-checked all entries, and resolved discrepancies through discussion with a third investigator.

### Reporting Quality Assessment

Two researchers with methodological training independently evaluated the reporting quality of the included studies using the TRIPOD statement [32,33]. This guideline covers 22 items spanning the title and abstract, introduction, methods, results, and discussion sections. In this review, each TRIPOD item was categorized as either reported, partially reported, or not reported. An item was considered reported if all key elements specified by TRIPOD were clearly described in the article, partially reported if only some elements were included or the description was incomplete, and not reported if the information was missing.

### Risk of Bias and Applicability Assessment

Two reviewers independently assessed the risk of bias using the PROBAST [34]. PROBAST comprises 4 domains—participants, predictors, outcome, and analysis—each with 20 signaling questions. Domain-level judgments were based on the responses to these questions. The overall risk of bias was rated as high if at least one domain was rated high risk, unclear if at least one domain was rated unclear and no domain was rated high risk, and low only when all domains were rated low risk [35]. Disagreements regarding TRIPOD or PROBAST were resolved through discussion, with a third reviewer acting as an arbitrator if needed. Interrater reliability statistics were not calculated because the independent preconsensus ratings were unavailable for analysis.

### Data Synthesis and Cross-Study Pattern Analysis

Given the substantial heterogeneity among the included studies, along with various biases and differences in modeling approaches, descriptive analyses and graphical representations were used to summarize the results. Adherence rates for each TRIPOD item were determined by dividing the number of studies meeting that specific item by the total number of studies for which the item was relevant. Overall adherence for each study was measured in a similar way. If a specific item was marked as “not applicable” for a study—such as the 6 items related to external validation (items 10c, 10e, 12, 13c, 17, and 19a), the 3 conditional items for model development (items 5c, 11, and 14b), or the 2 conditional items for model validation (items 10e and 17)—that item was excluded from the calculation of the TRIPOD compliance score. Additionally, since direct quantitative pooling of performance metrics was unsuitable due to heterogeneity in outcomes, modeling approaches, and validation strategies, model features and performance were summarized narratively using a structured comparison framework. Furthermore, cross-study patterns were examined narratively by categorizing studies based on predictor types, outcome domains, algorithm groups, validation approaches, and calibration metric reporting.

Because no meta-analysis of predictive performance was conducted and the included studies were highly diverse in outcomes, modeling approaches, and validation strategies, a formal statistical assessment of publication bias (eg, funnel plots or regression asymmetry tests) was not suitable. Instead, we qualitatively examined the possibility of reporting and dissemination bias by analyzing study characteristics, the completeness of reporting, and whether external validation and calibration assessments were present or absent.

## Results

### Study Selection

**Figure 1:**
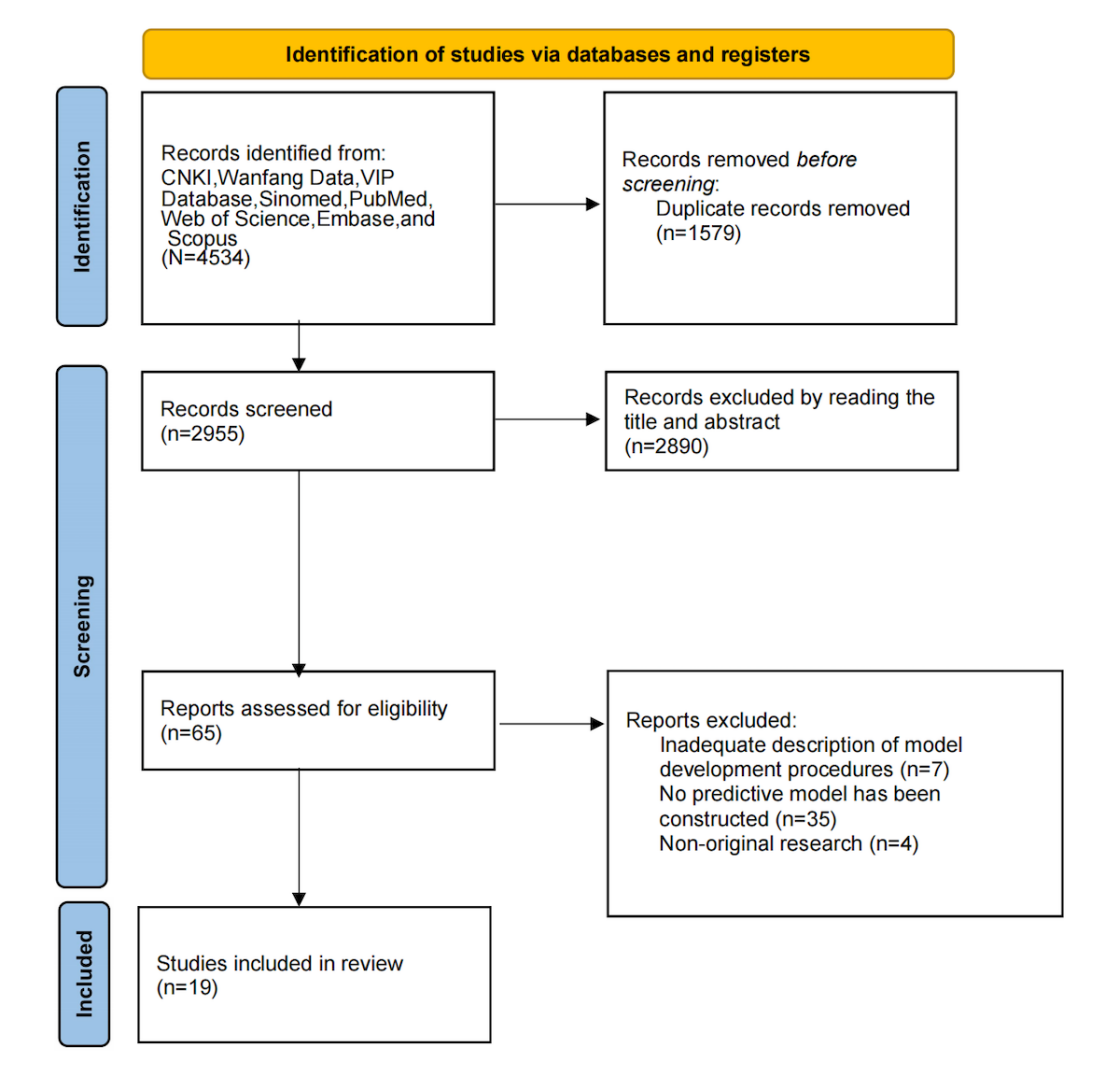
PRISMA (Preferred Reporting Items for Systematic Reviews and Meta-Analyses) flow diagram.

Initial searches across 8 databases yielded 4534 records. After removing duplicates, 2955 (65.2%) records remained. Title and abstract screening excluded 2890 (63.7%) records, leaving 65 (1.4%) articles for full-text review. After a comprehensive review of the full text, 19 (29.2%) studies were found to meet the inclusion criteria [18-26,36-45]. The study selection process is presented in Figure 1.

### Study and Model Characteristics

#### Overview

The characteristics of the included studies and models are summarized in Table 1, and detailed model characteristics are provided in Multimedia Appendix 1 [18-26,36-45]. Out of these 19 studies, only 1 (5.3%) [43] conducted external validation, using an independent prospective cohort (n=34) from a later period (July 2023-November 2023) as a temporal external validation set, distinct from the retrospective development dataset (January 2018-June 2023). A total of 16 (84.2%) studies [18,20-26,36,38-42,44,45] involved both model development and internal validation, while 2 (10.5%) studies [19,37] focused solely on model development. Nine (47.4%) studies included both surgical and conservative management approaches [19,22,24,26,37,41,43-45], whereas 3 (15.8%) studies modeled outcomes following anterior or posterior cervical approaches [18,23,43]. Additionally, 4 (21.1%) studies [20,36,38,40] did not specify treatment methods. All studies used more than 5 predictors. Regarding modeling algorithms, Maki et al [41] used 16 ML methods, Kitagawa et al [44] used 15, Zhang et al [25,43] applied 11 algorithms across 2 studies, and other studies used between 1 and 8 algorithms. These algorithms included CatBoost, LightGBM, random forest, gradient boosting, Extra Trees, XGBoost, k-nearest neighbors, Adaptive Boosting (AdaBoost), decision trees, and various regression methods (linear, ridge, least-angle, Bayesian ridge, lasso, Huber, and elastic net), along with support vector machines. Due to marked differences in prediction targets, modeling algorithms, sample sizes, and validation methods among the studies, a comparative analysis of model features and performance was performed in the following section.

**Table 1:** Characteristics of the included studies.

Study, country	Study design	Sample size (training set/validation set), n	Treatment	Outcome
Inoue et al [18] (2020), Japan	Retrospective cohort	165	ST^a^ and CT^b^	AIS^c^
Zhang et al [25] (2025), China	Retrospective cohort	134/34	CT	AIS and FIM^d^
Zhang et al [43] (2025), China	Retrospective cohort, prospective cohort	134/34	ST and CT	IANR^e^ score
Yoo et al [21] (2024), South Korea	Retrospective cohort	353	CT	FAC-DC^f^
Yoo et al [42] (2024), South Korea	Retrospective cohort	304/101	CT	FAC-DC
Tamburella et al [19] (2024), Italy	Retrospective cohort	951	ST and CT	SCIM^g^
Mascanzoni et al [37] (2024), Italy	Retrospective cohort	303	ST and CT	SCIM
Lin et al [23] (2025), China	Retrospective cohort	57/25	ST	AIS
Kishikawa et al [40] (2024), Japan	Retrospective cohort	60/20	NR^h^	AIS
Belliveau et al [20] (2016), United States	Retrospective cohort	3142	NR	FIM
DeVries et al [38] (2020), Canada	Retrospective cohort	862	NR	FIM
Draganich et al [24] (2024), United States	Retrospective cohort	3618/905	ST and CT	Walk 150 feet at home
Kapoor and Xu [36] (2023), United States	Retrospective cohort	18,737/2053	NR	AIS
Kato et al [39] (2024), Japan	Retrospective cohort	140/70	CT	SCIM
Kitagawa et al [44] (2025), Japan	Retrospective cohort	3703	ST and CT	AIS
Shimizu et al [45] (2025), United States	Retrospective cohort	247	ST and CT	AIS
Maki et al [41] (2024), Japan	Retrospective cohort	3122	ST, CT	FIM
Okimatsu et al [26] (2022), Japan	Retrospective cohort	215	ST and CT	AIS
Yang and Guo [22] (2022), China	Retrospective cohort	923/308	ST and CT	ADL^i^

^a^ST: surgical treatment.

^b^CT: conservative treatment.

^c^AIS: American Spinal Injury Association Impairment Scale.

^d^FIM: Functional Independence Measure.

^e^IANR: International Association of Neurorestoratology.

^f^FAC-DC: Functional Ambulation Category at discharge.

^g^SCIM: Spinal Cord Independence Measure.

^h^NR: not reported.

^i^ADL: activities of daily living.

Considering the considerable variability among the included studies, we conducted a structured narrative synthesis to uncover patterns rather than merely summarizing each model’s performance. Our cross-study comparison centered on predictor categories, outcome domains, algorithm families, validation methods, and the reporting of performance metrics, including calibration.

#### Predictor Groups

The predictors used in various studies can be generally categorized into four groups: (1) demographic and baseline patient attributes, such as age and sex; (2) indicators of neurological and clinical severity, including AIS grade and functional status at the start; (3) treatment-related factors such as surgical versus conservative management and perioperative details; and (4) features derived from imaging or other high-dimensional data, especially radiomics-based variables. Most (13/19, 68.4%) studies [18-22,24,36-41,44] used multivariable predictor sets that combined demographic and clinical factors, while a smaller number (5/19, 26.3%) [23,25,26,43,45] included imaging features or multimodal data. This pattern suggests that current SCI prognostic models primarily rely on routinely collected clinical data, whereas imaging-focused models constitute a more specialized subset.

#### Variability in Outcomes

There was considerable heterogeneity in the predicted outcomes, including neurological recovery, functional independence, gait recovery, ADL, discharge walking ability, and postoperative recovery. The studies used various measures, including AIS, SCIM, FIM, Functional Ambulation Category at discharge (FAC-DC), ADL, walking ability, and International Association of Neurorestoratology (IANR) score, as their primary outcomes. Outcomes related to AIS were the most frequently reported, followed by SCIM and FIM. Besides differences in outcome scales, the timing and clinical relevance of these outcomes varied, ranging from short-term neurological improvements to functional status at discharge and beyond. This variability complicates direct comparisons of model performance across different studies.

#### Algorithm Families

The studies also showed distinct clustering based on algorithm family. One group [21,23,42] mainly used regression-based or traditional statistical models, such as logistic regression, linear regression, ridge regression, and lasso. A second group [18,21,25,43] used ML methods based on trees or kernels, including random forest, decision trees, support vector machines, XGBoost, LightGBM, gradient boosting, and related ensemble techniques. A third group [19,20,26,37,40] used neural network or deep learning approaches, such as artificial neural networks, convolutional neural networks, recurrent neural networks, and deep learning–based radiomics. Tree-based and ensemble algorithms were among the most frequently tested, often compared with regression methods within the same dataset. Nonetheless, no single algorithm family consistently outperformed others in all studies, due to notable variations in outcomes, predictor sets, and validation protocols.

#### Validation Patterns

Validation strategies varied across the studies. Of the 19 studies, 2 (10.5%) [19,37] focused solely on model development, while most (16/19, 84.2%) combined model development with internal validation methods, such as data partitioning or cross-validation [18,20-26,36,38-42,44,45]. Only 1 (5.3%) study [43] performed external validation using an independent temporal dataset. Consequently, the evidence base is mainly composed of internally validated studies, with limited evaluation of how well these models generalize to other settings.

#### Patterns in Performance Reporting

The evaluation of model performance primarily focused on discrimination metrics. Most classification studies reported measures such as area under the receiver operating characteristic curve, accuracy, sensitivity, and specificity, whereas regression studies reported root mean square error, mean absolute error, coefficient of determination (*R*^2^), mean squared error, or root mean squared logarithmic error more frequently. Conversely, calibration was seldom reported in a structured way, with formal calibration metrics or plots largely absent. Only 5.3% (1/19) of study [23] used decision curve analysis, but these do not replace calibration assessment. This pattern suggests that the current literature emphasizes discrimination over agreement between predicted and observed outcomes, thereby hampering the assessment of clinical usefulness.

Overall, these cross-study patterns indicate that the literature on ML-based SCI prognostic models is marked by both methodological variety and common structural imbalances. These include frequent use of mixed clinical predictors, marked variability in outcome definitions, a focus on tree-based and ensemble algorithms, reliance on internal validation, and limited calibration reporting. These patterns are important to consider when evaluating differences in model performance. A structured comparison of study features is provided in Table 1 and Multimedia Appendix 1 [18-26,36-45].

### Reporting Quality

#### Overview

Regarding reporting completeness, of the 19 studies, 7 (36.8%) [21,23-25,37,40,41] achieved at least 70% adherence across all TRIPOD domains, including title and abstract, background and objectives, methods, results, discussion, and supplementary information. Conversely, 2 (10.5%) studies [36,45] reported adherence of less than 60%. The adherence rates for each TRIPOD item are detailed in Multimedia Appendix 2. Overall, adherence per study ranged from 54.8% (17/31) to 81.1% (30/37), with a median of 74.2% (IQR 64.5%-77.4%). Additionally, 7 (36.8%) studies [18,20,22,26,38,39,42] met or exceeded the 60% adherence threshold.

#### Title and Abstract (Items 1-2) and Background and Objectives (Items 3)

Of the 19 studies, 14 (73.7%) [18-21,23,24,36-38,40,42-45] fully reported the requirements for titles and abstracts. Five (26.3%) studies [25,26,39,41,43] did not explicitly clarify whether they focused on model development, validation, or both, although most (14/19, 73.7%) included target populations and outcomes in their titles. A minority of the descriptions included predictors in the abstracts. All studies provided detailed descriptions of their research backgrounds.

#### Methods (Items 4-12)

All studies reported data sources, outcome definitions, assessment methods, timing for predictors, and model performance metrics. A total of 14 (73.7%) studies [19-21,23-25,36-41,44,45] specified key study dates; 13 (68.4%) studies [19,20,23-26,37-41,43,45] detailed participant inclusion and exclusion criteria. Treatment information was provided in 5 (26.3%) studies [19,23,42-44]. Only 2 (10.5%) studies [41,44] described procedures for blinded outcome assessment unaffected by predictor knowledge. Blinded predictor assessment was used in 4 (21.1%) studies [18,21,43,44]. Six (31.6%) studies [22,24,25,38,39,41] documented the handling of missing data using imputation methods. Risk stratification criteria were clearly defined in 4 (21.1%) studies [23,40,42,43]. One (5.3%) study [45] did not report critical setting elements and predictor preprocessing. Another [18] omitted details on full model development procedures. None of the studies justified their sample size. Zhang et al [43], in an external validation study, reported the calculation methods for predictors and noted discrepancies in settings, eligibility criteria, outcomes, and predictors compared with the development dataset, but did not include details on model updating.

#### Results (Items 13-17)

All studies (19/19, 100%) presented performance metrics for the prediction models. Participant flow diagrams were included in 16 (84.2%) studies [19-26,36-41,43,44]. Sixteen (84.2%) studies [18-26,37-41,44,45] provided details on the number of participants and outcome events. Seven (36.8%) studies [19,21-23,37,42,43] reported unadjusted associations between potential predictors and outcomes. Full model specifications were available in 10 (52.6%) studies [18,19,21,22,25,26,37,41,43,44], while instructions for applying the models were given in 10 (52.6%) studies [18,19,21,22,25,26,41-44]. Only 1 (5.3%) study [42] lacked basic demographic information. Although Zhang et al [43] performed external validation, they did not specify any results related to model updating.

#### Discussion (Items 18-20) and Supplementary Information (Items 21-22)

All studies acknowledged limitations and provided overall interpretations of their findings, covering study objectives, limitations, results from similar research, and other relevant evidence. In total, 12 (63.2%) studies [18-21,24,37,38,40-43,45] addressed clinical applicability and implications for future research. Supplementary resource availability was noted in 7 (36.8%) studies [23-25,40,43-45]. Funding sources and the roles of funders were disclosed in 13 (68.4%) studies [18-20,23,24,26,37,39-44]. Zhang et al [43] reported results based on development data and other validation datasets.

### Risk of Bias and Applicability Assessment

The risk of bias and applicability of the included studies were assessed using PROBAST, with detailed results shown in Table 2. All 19 studies were rated as having a high overall risk of bias. In PROBAST, judgments were based on domain-specific signaling questions and the overall pattern of methodological limitations across studies. In the participants’ domain, concerns mainly focused on the use of retrospectively assembled cohorts with limited reporting of eligibility criteria, patient selection procedures, or data source consistency, rather than on the retrospective design itself. For predictors, 10 (52.6%) studies [19,20,22,23,36-40,42] were rated as high risk due to possible inconsistencies in predictor assessment and data collection methods across centers, as well as the risk of bias from outcome awareness during measurement. In the outcome domain, 17 (89.5%) studies [19,20,22-26,36-45] were judged to be at high risk of bias because outcome assessment procedures were insufficiently reported and may have been influenced by knowledge of predictor information. In the analysis domain, some studies (n=2, 10.5%) did not meet commonly used sample size rules of thumb for prediction model research, such as ≥20 events per variable during model development [46] and at least 100 events and 100 nonevents for external validation [47]. Two (10.5%) studies [23,40] had insufficient modeling samples, and 4 (21.1%) studies [25,39,40,43] had validation samples below 100. Predictor selection was based solely on literature or clinical experience in 5 (26.3%) studies [21,23,36,41,42], while selection methods were unreported in 2 (10.5%) studies [26,45]. Missing data handling was inadequate in 13 (68.4%) studies [18-21,23,26,36,37,40,42-45]. Overall applicability concerns were deemed low across the included studies, consistent with Table 2, indicating that the review questions, target populations, predictors, and outcomes of the included studies broadly align with the scope of this review.

**Table 2:** Assessment of study bias risk and applicability.

Study	Risk of bias				Applicability			Overall evaluation	
	Participants	Predictors	Outcome	Analysis	Participants	Predictors	Outcome	Risk of bias	Applicability
Inoue et al [18]	High	Low	Low	High	Low	Low	Low	High	Low
Zhang et al [25]	High	Low	High	High	Low	Low	Low	High	Low
Zhang et al [43]	High	Low	High	High	Low	Low	Low	High	Low
Yoo et al [21]	High	Low	Low	High	Low	Low	Low	High	Low
Yoo et al [42]	High	High	High	High	Low	Low	Low	High	Low
Tamburella et al [19]	High	High	High	High	Low	Low	Low	High	Low
Mascanzoni et al [37]	High	High	High	High	Low	Low	Low	High	Low
Lin et al [23]	High	High	High	High	Low	Low	Low	High	Low
Kishikawa et al [40]	High	High	High	High	Low	Low	Low	High	Low
Belliveau et al [20]	High	High	High	High	Low	Low	Low	High	Low
DeVries et al [38]	High	High	High	High	Low	Low	Low	High	Low
Draganich et al [24]	High	Low	High	High	Low	Low	Low	High	Low
Kapoor and Xu [36]	High	High	High	High	Low	Low	Low	High	Low
Kato et al [39]	High	High	High	High	Low	Low	Low	High	Low
Kitagawa et al [44]	High	Low	High	High	Low	Low	Low	High	Low
Shimizu et al [45]	High	Low	High	High	Low	Low	Low	High	Low
Maki et al [41]	High	Low	High	High	Low	Low	Low	High	Low
Okimatsu et al [26]	High	Low	High	High	Low	Low	Low	High	Low
Yang and Guo [22]	High	High	High	High	Low	Low	Low	High	Low

## Discussion

### Principal Findings

This systematic review found that ML-based prognostic prediction models for adults with SCI remain methodologically promising but clinically immature. Across 19 studies, TRIPOD adherence was incomplete, with a median of 15 of 22 TRIPOD items (74.2%) completed, and all studies were judged to have a high overall risk of bias. The main weaknesses were insufficient justification of sample size, incomplete handling of missing data, limited reporting of blinded predictor and outcome assessment, scarce calibration assessment, and very limited external validation. Only 1 (5.3%) of 19 studies externally validated a model. These findings indicate that most models should be viewed as exploratory rather than ready for routine clinical implementation, which is consistent with recent SCI-focused evidence showing that, although ML models may predict SCI prognosis with relative accuracy, larger datasets, stronger validation, and more rigorous model development remain necessary before clinical implementation [48,49].

### Methodological and Reporting Limitations

The high overall risk of bias observed in this review should not be interpreted as meaning that retrospective prediction model studies are inherently unreliable. Rather, the PROBAST judgments reflected multiple modifiable methodological limitations, particularly in the analysis domain. None of the included studies justified their sample size, 13 (68.4%) of 19 studies had inadequate handling of missing data, and several (n=4, 21.1%) studies tested multiple algorithms without sufficient evidence that overfitting was adequately controlled. In several (n=4, 21.1%) studies [20,22,36,38], concerns also arose from insufficiently described participant selection procedures or limited data sources, which may have reduced representativeness and increased the risk of selection bias.

Prospective studies require standardized, multidimensional clinical data collected over clinically meaningful follow-up periods, which is resource-intensive and difficult to implement in SCI prognostic modeling [48,50,51]. As a result, most included studies relied on retrospective datasets. Although retrospective data can be useful for early model development, they are often affected by small sample sizes, heterogeneous treatment pathways, incomplete follow-up, and missing baseline or outcome data. For example, Lin et al [23] included only 82 eligible patients, and Kishikawa et al [40] enrolled 80 patients without clearly defined inclusion or exclusion criteria, both of which may have increased the risk of biased or unstable model estimates.

Incomplete adherence to prediction model reporting and appraisal standards was another important limitation, particularly considering recent PROBAST+AI guidance, which emphasizes quality, risk of bias, and applicability assessment for prediction models developed using regression or AI methods [52]. The included studies did not fully follow TRIPOD and PROBAST recommendations. Common problems included insufficient reporting of treatment details, lack of blinded predictor or outcome assessment, incomplete description of missing-data methods, unclear risk stratification criteria, inadequate reporting of unadjusted predictor-outcome associations, and limited availability of model implementation details or supplementary materials. Similar problems have been reported in other reviews of clinical prediction models, in which most included studies were judged to have a high risk of bias because of methodological flaws, incomplete outcome reporting, or small sample sizes [53,54]. These findings suggest that the methodological limitations identified in this review are not unique to SCI but reflect broader challenges in ML-based prediction research.

External validation was particularly limited. Only one included study [43] conducted external validation using an independent temporal cohort. This is problematic because internal validation alone cannot fully assess whether a model will generalize to different institutions, patient populations, rehabilitation pathways, or clinical settings [50,55]. Models without external validation may show optimistic performance in the development dataset but perform less well when applied in real-world clinical practice. Therefore, the current evidence base remains insufficient to support broad implementation of these models, particularly because clinical prediction models generally require external validation, model updating when performance changes across settings, and implementation evaluation before routine clinical use [51].

### Substantial Interstudy Heterogeneity

Substantial heterogeneity was observed across studies in outcome measures, predictor selection, modeling algorithms, validation strategies, and performance metrics. The predicted outcomes included neurological recovery, functional independence, gait recovery, ADL, discharge walking ability, and postoperative recovery. Different studies used different outcome scales, including AIS, SCIM, FIM, FAC-DC, ADL, walking ability, and IANR. The timing of outcome assessment also varied, ranging from short-term neurological improvement to functional status at discharge or later follow-up. This heterogeneity prevents direct quantitative comparison of model performance and limits the ability to identify which modeling approach is most suitable for SCI prognosis.

Algorithm selection also varied widely. Some studies used traditional regression-based models, whereas others used tree-based methods, ensemble algorithms, neural networks, deep learning, or radiomics-based approaches. Several studies [41,44] tested 15 to 16 algorithms, which may increase the risk of overfitting or selective emphasis on the best-performing model, particularly when sample sizes are limited. At present, no single algorithm family can be considered consistently superior across SCI prognostic tasks because the available studies differ substantially in clinical context, outcome definitions, predictor sets, and validation methods.

### Clinical Applicability and Implementation Barriers

Beyond methodological quality, the clinical usefulness of current SCI prognostic models remains limited. For a model to be clinically valuable, it should answer a clearly defined clinical question, use predictors available at the time of decision-making, provide outputs that clinicians can interpret, and demonstrate acceptable performance beyond the original development dataset [50,56-59]. Most included studies did not fully meet these conditions.

First, many models were developed using retrospective data from single centers or limited multicenter datasets, raising concerns about generalizability across different health care systems, rehabilitation pathways, and patient populations [60]. Second, some models relied on complex pipelines, imaging-derived features, or multiple algorithms without clearly demonstrating bedside feasibility. A model may perform well internally but still have limited practical value if it depends on specialized software, uncommon image-processing procedures, or predictors that are not routinely available in daily clinical care.

Third, interpretability is especially important in SCI care. Prognostic predictions are used not only to support clinicians’ decisions but also to communicate expected neurological recovery, functional prognosis, rehabilitation goals, and discharge planning to patients and families [61-63]. Models that lack clear explanations may have limited acceptance, even if they show good discrimination. Fourth, prediction errors may have meaningful clinical consequences. Overly optimistic predictions could misguide rehabilitation planning and patient counseling, whereas overly pessimistic predictions could lead to unnecessary restrictions in therapy intensity or referral decisions [64]. Therefore, clinical implementation requires not only discrimination or accuracy but also calibration, transparency, interpretability, robustness, usability, and assessment of decision consequences [65].

### Comparison Between ML and Traditional Models

Although some included studies reported better discrimination or accuracy for certain ML models than for conventional statistical approaches, the current evidence does not support a general conclusion that ML consistently outperforms traditional models in SCI prognostic prediction. Direct comparisons were limited, and when available, they were usually based on individual datasets with different outcomes, predictor sets, preprocessing methods, and validation strategies. Because most studies relied only on internal validation and all were judged to have a high overall risk of bias, any apparent performance advantage of ML should be interpreted cautiously.

A more balanced interpretation is that ML may be useful in specific scenarios, particularly when modeling complex nonlinear relationships, high-dimensional predictors, imaging-derived features, or multimodal clinical data. For example, radiomics-based or ensemble techniques may be promising for selected postoperative or imaging-focused prediction tasks [25,43]. However, traditional regression models may remain appropriate when sample sizes are small, predictor sets are limited, and interpretability is a priority. Nomogram-based approaches may also offer practical value because they can translate complex statistical models into visual tools that support individualized risk estimation [25]. Future studies should conduct transparent and predefined comparisons between ML and conventional models within the same clinical context, using rigorous external validation, calibration assessment, and clinical utility evaluation [66].

### Implications for Future Research

Future research should prioritize the development of streamlined, clinically meaningful, and externally validated prediction models. This review examined 42 prediction models using diverse predictors, including demographic characteristics, disease-related variables, imaging features, and treatment-related factors, reflecting the multifactorial nature of SCI prognosis. However, adding too many predictors can increase computational burden, reduce model stability, and make clinical implementation more difficult [50,67]. Future models should therefore use careful variable selection, simpler model structures when appropriate, regularization methods to reduce overfitting, and validation protocols that reflect real clinical settings [67,68].

Standardization is also essential. Future studies should develop core outcome sets for SCI prognostic modeling, clearly define outcome timing, and consider more granular outcomes, such as segmental motor recovery when clinically appropriate, rather than relying only on broad functional scales or aggregate motor scores [69]. Multicenter prospective cohorts are needed to increase sample size, improve representativeness, and reduce the effect of single-center practice patterns. Researchers should justify sample size requirements, report missing-data methods with clear methodological reasoning, assess calibration, and provide full model specifications or accessible implementation tools. External validation should include temporal, geographic, or multicenter validation, and model updating should be considered when performance changes across settings or over time.

### Study Limitations

This review has several limitations. First, substantial heterogeneity in predictors, modeling algorithms, outcome measures, follow-up times, and performance metrics prevented quantitative pooling or direct comparison of model performance. Second, although we used TRIPOD and PROBAST to systematically assess reporting quality and risk of bias, these assessments depended on the information reported in the original studies; therefore, some judgments may have been influenced by incomplete reporting rather than by confirmed methodological flaws. Third, although TRIPOD and PROBAST assessments were conducted independently by 2 reviewers and disagreements were resolved by consensus, interrater reliability measures, such as Cohen kappa, were not calculated because the independent preconsensus ratings were unavailable. This may limit the transparency of the assessment process. Finally, we did not formally evaluate publication bias because quantitative pooling was not performed and the included studies were highly heterogeneous; however, reporting and dissemination bias may still exist and should be considered when interpreting the findings.

### Conclusions

ML shows methodological promise for prognostic prediction in SCI, particularly when complex clinical, imaging, or high-dimensional data are available. However, current evidence remains exploratory because of incomplete reporting, high risk of bias, heterogeneous outcomes, limited calibration assessment, and scarce external validation. Future studies should use standardized SCI-specific outcome frameworks, transparent reporting, appropriate sample size justification, robust external validation, and explicit assessment of clinical usefulness before these models are implemented in routine practice.

## Appendix

Multimedia Appendix 1 Detailed model characteristics of the included studies.

Multimedia Appendix 2 Adherence to individual TRIPOD items across the included studies.

## Abbreviations

AdaBoost: Adaptive Boosting
ADL: activities of daily living
AI: artificial intelligence
AIS: American Spinal Injury Association Impairment Scale
CHARMS: Critical Appraisal and Data Extraction for Systematic Reviews of Prediction Modeling Studies
FAC-DC: Functional Ambulation Category at discharge
FIM: Functional Independence Measure
LightGBM: Light Gradient Boosting Machine
MeSH: Medical Subject Headings
ML: machine learning
PRISMA: Preferred Reporting Items for Systematic Reviews and Meta-Analyses
PROBAST: Prediction Model Risk of Bias Assessment Tool
QUADAS-2: Quality Assessment of Diagnostic Accuracy Studies 2
SCI: spinal cord injury
SCIM: Spinal Cord Independence Measure
TRIPOD: Transparent Reporting of a Multivariable Prediction Model for Individual Prognosis or Diagnosis
XGBoost: Extreme Gradient Boosting
