# Supplementary material for: Machine Learning–Based Prognostic Models for Functional Outcomes in Spinal Cord Injury: Systematic Review
Source: JMIR Med Inform. 2026 Jun 23;14:e84980. doi: 10.2196/84980 (PMC13342815; doi:10.2196/84980)
Supplement: Multimedia Appendix 1 [file medinform_v14i1e84980_app1.docx]

| Author  (year),  country | Model type | Modeling method | Validation method | Performance metrics | TRIPOD adherence, % |
| --- | --- | --- | --- | --- | --- |
| Inoue et al [18]  (2020), Japan | Development with internal validation | XGBoost^a^, LogReg^b^, DT^c^ | 8-fold cross-validation | ROC^d^, AUC^e^, ACC^f^, True Positive Rate, False Positive Rate | 64.5% |
| Zhang et al [25] (2025), China | Development with internal validation | LogReg, NB^g^, SVM^h^, KNN^i^, RF^j^, Extremely Randomized Tree Classifier, XGBoost, LightGBM^k^, Gradient Boosting Classifier, ABC^l^, MLP^m^ classifier | Data partitioning, 5-fold cross-validation | AUC, ACC, Specificity, Sensitivity | 80.0% |
| Zhang et al [43] (2025), China | Development with internal and external validation | LogReg, NB, SVM, KNN, RF, Extremely Randomized Tree Classifier, XGBoost; LightGBM, Gradient Boosting Classifier, ABC, MLP classifier | 10-fold cross-validation, external validation set | ROC, AUC, Sensitivity, Specificity, ACC | 81.0% |
| Yoo et al [21] (2024), South Korea | Development with internal validation | LinReg^n^, RF, DT, SVM | Data partitioning, 5-fold cross-validation | AUC, ACC, Specificity, Sensitivity | 74.2% |
| Yoo et al [42] (2024), South Korea | Development with internal validation | RNN^o^, LinReg, Ridge^p^, Lasso^q^ | Data partitioning | RMSE^r^ | 64.5% |
| Tamburella et al [19] (2024), Italy | Development only | ANN, LinReg | NR | Raw Importance, Normalized Importance | 80.6% |
| Mascanzoni et al [37] (2024), Italy | Development only | ANN | NR | RMSE | 74.2% |
| Lin et al [23] (2025), China | Development with internal validation | LogReg, SVM, RF, NB, MLP classifier, LightGBM | Data partitioning, 10-fold cross-validation | AUC, Decision Curve Analysis, Specificity, Sensitivity, ACC | 77.4% |
| Kishikawa et al [40] (2024), Japan | Development with internal validation | Multiple LinReg, ANN | Data partitioning, 10-fold cross-validation | ACC | 74.2% |
| Belliveau et al [20] (2016), United States | Development with internal validation | ANN, LogReg | Data partitioning, 10-fold cross-validation | ROC, AUC, ACC, Specificity, Sensitivity | 67.7% |
| DeVries et al [38] (2020), Canada | Development with internal validation | Unsupervised machine learning algorithms, LogReg, van Middendorp logistic regression model, Hicks clinical prediction rule | 10-fold cross-validation | AUC, ACC, Specificity, Sensitivity | 67.7% |
| Draganich et al [24] (2024), United States | Development with internal validation | van Middendorp logistic regression model, Hicks clinical prediction rule, EN^s^, LogReg, gradient boosting machine, ANN | Data partitioning, 10-fold cross-validation | AUC, ACC, Specificity, Sensitivity | 74.2% |
| Kapoor et al [36] (2023), United States | Development with internal validation | Ridge, SVM, EN, LogReg, Ensemble Models (EN, KNN, RF), CNN^t^, RF, NB | Data partitioning, 10-fold cross-validation | ACC | 54.8% |
| Kato et al [39] (2024), Japan | Development with internal validation | ANN, RF, SVM, gradient boosting, Ridge-based stacked ensemble | Data partitioning, 5-fold cross-validation | RMSE, R^2u^, MAE | 61.3% |
| Kitagawa et al [44] (2025), Japan | Development with internal validation | GBR, LightGBM, RF, Extra Trees, LinReg, Ridge Regression, Bayesian Ridge, Least Angle Regression, Huber, AdaBoost Regressor, KNN, EN, Lasso, Passive Aggressive Regressor, Orthogonal Matching Pursuit, Dummy Regressor | 10-fold cross-validation | RMSLE^v^, MSE^w^, RMSE, R^2^ | 80.6% |
| Shimizu et al [45] (2025), United States | Development with internal validation | Lightweight Gradient Boosting Machine | 5-fold cross-validation | ACC, Sensitivity, Prec^x^, AUC | 58.1% |
| Maki et al [41] (2024), Japan | Development with internal validation | CatBoost^y^, LightGBM, RF, GBR, Extra Trees, XGBoost, KNN, LinReg, Ridge, Lasso, EN, Huber, AdaBoost Regressor, Orthogonal Matching Pursuit, DT | 10-fold cross-validation | AUC, ROC, ACC, R^2^, Mean Absolute Error, RMSE | 77.4% |
| Okimatsu et al [26] (2022), Japan | Development with internal validation | CNN, RF, Deep Learning-based Radiomics | 5-fold cross-validation | ACC, Prec, Sensitivity, F value | 61.3% |
| Yang et al [22] (2022), China | Development with internal validation | RF, Harris Hawks Optimizer, Harris Hawks Optimizer–Random Forest model, Bayesian Ridge Regression, XGBoost, SVM | Data partitioning, 10-fold cross-validation | Mean Absolute Error, RMSE, R^2^ | 64.5% |

^a^XGBoost: Extreme Gradient Boosting.

^b^LogReg: Logistic Regression.

^c^DT: Decision Tree.

^d^ROC: Receiver Operating Characteristic Curve.

^e^AUC: Area Under the (ROC) Curve.

^f^ACC: Accuracy.

^g^NB: Naive Bayes (Classifier).

^h^SVM: Support Vector Machine.

^i^KNN: K-Nearest Neighbor.

^j^RF: Random Forest.

^k^LightGBM: Light Gradient Boosting Machine.

^l^ABC: AdaBoost Classifier.

^m^MLP: Multi-Layer Perceptron.

^n^LinReg: Linear Regression.

^o^RNN: Recurrent Neural Network.

^p^Ridge: Ridge Regression.

^q^Lasso: Least Absolute Shrinkage and Selection Operator.

^r^RMSE: Root Mean Square Error.

^s^EN: Elastic Net Regression.

^t^CNN: Convolutional Neural Network.

^u^R^2^: Coefficient of Determination.

^v^RMSLE: Root Mean Squared Logarithmic Error.

^w^MSE: Mean Square Error.

^x^Prec: Precision.

^y^CatBoost: Categorical Boosting.

**Reference**

18. Inoue T, Ichikawa D, Ueno T, Cheong M, Inoue T, Whetstone WD, et al. XGBoost, a Machine Learning Method, Predicts Neurological Recovery in Patients with Cervical Spinal Cord Injury. Neurotrauma Rep. 2020;1(1):8-16. doi:10.1089/neur.2020.0009

19. Tamburella F, Lena E, Mascanzoni M, Iosa M, Scivoletto G. Harnessing Artificial Neural Networks for Spinal Cord Injury Prognosis. J Clin Med. 2024;13(15). doi:10.3390/jcm13154503

20. Belliveau T, Jette AM, Seetharama S, Axt J, Rosenblum D, Larose D, et al. Developing Artificial Neural Network Models to Predict Functioning One Year After Traumatic Spinal Cord Injury. Arch Phys Med Rehabil. 2016;97(10):1663-8.e3. doi:10.1016/j.apmr.2016.04.014

21. Yoo HJ, Koo B, Yong CW, Lee KS. Prediction of gait recovery using machine learning algorithms in patients with spinal cord injury. Medicine (United States). 2024;103(23). doi:10.1097/MD.0000000000038286

22. Yang F, Guo X. Research on Rehabilitation Effect Prediction for Patients with SCI Based on Machine Learning. World Neurosurg. 2022;158:e662-e74. doi:10.1016/j.wneu.2021.11.040

23. Lin F, Wang K, Lai M, Wu Y, Chen C, Wang Y, et al. Multicenter study on predicting postoperative upper limb muscle strength improvement in cervical spinal cord injury patients using radiomics and deep learning. Sci Rep. 2025;15(1):5805. doi:10.1038/s41598-024-72539-0

24. Draganich C, Anderson D, Dornan GJ, Sevigny M, Berliner J, Charlifue S, et al. Predictive modeling of ambulatory outcomes after spinal cord injury using machine learning. Spinal Cord. 2024;62(8):446-53. doi:10.1038/s41393-024-01008-2

25. Zhang Z, Li N, Ding Y, Cheng H. An integrative nomogram based on MRI radiomics and clinical characteristics for prognosis prediction in cervical spinal cord Injury. Eur Spine J. 2025;34(3):1164-76. doi:10.1007/s00586-024-08609-8

26. Okimatsu S, Maki S, Furuya T, Fujiyoshi T, Kitamura M, Inada T, et al. Determining the short-term neurological prognosis for acute cervical spinal cord injury using machine learning. Journal of Clinical Neuroscience. 2022;96:74-9. doi:10.1016/j.jocn.2021.11.037

36. Kapoor D, Xu C. Spinal Cord Injury AIS Predictions Using Machine Learning. eNeuro. 2023;10(1). doi:10.1523/ENEURO.0149-22.2022

37. Mascanzoni M, Luciani A, Tamburella F, Iosa M, Lena E, Di Fonzo S, et al. The Role of Psychological Variables in Predicting Rehabilitation Outcomes After Spinal Cord Injury: An Artificial Neural Networks Study. J Clin Med. Dec 2024;13(23). PMID:39685573 doi:10.3390/jcm13237114

38. DeVries Z, Hoda M, Rivers CS, Maher A, Wai E, Moravek D, et al. Development of an unsupervised machine learning algorithm for the prognostication of walking ability in spinal cord injury patients. Spine J. 2020;20(2):213-24. doi:10.1016/j.spinee.2019.09.007

39. Kato C, Uemura O, Sato Y, Tsuji T. Functional Outcome Prediction After Spinal Cord Injury Using Ensemble Machine Learning. Arch Phys Med Rehabil. 2024;105(1):95-100. doi:10.1016/j.apmr.2023.08.011

40. Kishikawa J, Kobayakawa K, Saiwai H, Yokota K, Kubota K, Hayashi T, et al. Verification of the Accuracy of Cervical Spinal Cord Injury Prognosis Prediction Using Clinical Data-Based Artificial Neural Networks. J Clin Med. 2024;13(1). doi:10.3390/jcm13010253

41. Maki S, Furuya T, Inoue T, Yunde A, Miura M, Shiratani Y, et al. Machine Learning Web Application for Predicting Functional Outcomes in Patients With Traumatic Spinal Cord Injury Following Inpatient Rehabilitation. J Neurotrauma. 2024;41(9-10):1089-100. doi:10.1089/neu.2022.0383

42. Yoo HJ, Lee KS, Koo B, Yong CW, Kim CW. Deep Learning-Based Prediction Model for Gait Recovery after a Spinal Cord Injury. Diagnostics. 2024;14(6). doi:10.3390/diagnostics14060579

43. Zhang Z, Li N, Ding Y, Sun H, Cheng H. Establishment and validation of a ResNet-based radiomics model for predicting prognosis in cervical spinal cord injury patients. Sci Rep. 2025;15(1):9163. doi:10.1038/s41598-025-94358-7

44. Kitagawa K, Maki S, Furuya T, Shiratani Y, Nagashima Y, Maruyama J, et al. Development of a machine learning model and a web application for predicting neurological outcome at hospital discharge in spinal cord injury patients. Spine J. 2025. doi:10.1016/j.spinee.2025.01.005

45. Shimizu T, Inomata K, Suda K, Matsumoto Harmon S, Komatsu M, Ota M, et al. A multimodal machine learning model integrating clinical and MRI data for predicting neurological outcomes following surgical treatment for cervical spinal cord injury. Eur Spine J. 2025. doi:10.1007/s00586-025-08873-2
