# Supplementary material for: Machine Learning–Based Prognostic Models for Functional Outcomes in Spinal Cord Injury: Systematic Review
Source: JMIR Med Inform. 2026 Jun 23;14:e84980. doi: 10.2196/84980 (PMC13342815; doi:10.2196/84980)
Supplement: Multimedia Appendix 2 [file medinform_v14i1e84980_app2.docx]

| TRIPOD item | Section | Number of studies reported, n/N | Adherence, % |
| --- | --- | --- | --- |
| 1 | Title | 14/19 | 73.7% |
| 2 | Abstract | 19/19 | 100% |
| 3a | Background | 19/19 | 100% |
| 3b | Objectives | 18/19 | 94.7% |
| 4a | Design/data | 19/19 | 100% |
| 4b | Datas | 14/19 | 73.7% |
| 5a | Setting | 18/19 | 94.7% |
| 5b | Eligibility | 12/19 | 63.1% |
| 5c | Treatment details | 5/19 | 26.3% |
| 6a | Outcome | 19/19 | 100% |
| 6b | Outcome blinding | 2/19 | 10.5% |
| 7a | Predictors | 19/19 | 100% |
| 7b | Predictor blinding | 4/19 | 21.1% |
| 8 | Sample size | 0/19 | 0 |
| 9 | Missingdata | 6/19 | 31.6% |
| 10a | Predictor handing | 18/19 | 94.7% |
| 10b | Model specification | 18/19 | 94.7% |
| 10c | Prediction calculation | 1/19 | 5.3% |
| 10d | Model performance | 19/19 | 100% |
| 10e | Model updating | 0/19 | 0 |
| 11 | Risk groups | 4/19 | 21.1% |
| 12 | D/V data description | 1/19 | 5.3% |
| 13a | Participants flow | 16/19 | 84.2% |
| 13b | Participants characteristics | 18/19 | 94.7% |
| 13c | D/V data comparison | 1/19 | 5.3% |
| 14a | Numbers in analysis | 16/19 | 84.2% |
| 14b | Unadjusted associations | 7/19 | 36.8% |
| 15a | Presentation of full model | 10/19 | 52.6% |
| 15b | Model explanation | 10/19 | 52.6% |
| 16 | Model performance | 19/19 | 100% |
| 17 | Results of model updating | 0/19 | 0 |
| 18 | Limitations | 19/19 | 100% |
| 19a | Vakidation interpretation | 1/19 | 5.3% |
| 19b | Overall interpretation | 19/19 | 100% |
| 20 | Implications | 12/19 | 63.2% |
| 21 | Supplementary | 7/19 | 36.8% |
| 22 | Funding | 13/19 | 68.4% |
